# Clinical Outcomes for Systemic Corticosteroids Versus Vincristine in Treating Kaposiform Hemangioendothelioma and Tufted Angioma

**DOI:** 10.1097/MD.0000000000003431

**Published:** 2016-05-20

**Authors:** Xiaohan Liu, Jiaying Li, Xinhua Qu, Weili Yan, Ling Zhang, Shanyong Zhang, Chi Yang, Jiawei Zheng

**Affiliations:** From the Department of Oral Surgery (XL, CY, SZ); and Department of Oral-Maxillofacial Head and Neck Surgery (JZ, LZ), Shanghai Ninth People's Hospital, College of Stomatology, Shanghai Jiao Tong University School of Medicine, Shanghai Key Laboratory of Stomatology, Shanghai, China; Key Laboratory of Orthopedic Implant (XQ), Shanghai Ninth People's Hospital, Shanghai Jiao Tong University School of Medicine; Jining Medical University (JL), Jining, Shandong Province; Pudong Institute of Preventive Medicine (WY), Fudan University, Shanghai, China.

## Abstract

A meta-analysis was performed to evaluate the efficacy and safety of systemic corticosteroids versus those of vincristine in the treatment of kaposiform hemangioendothelioma (KHE) and tufted angioma (TA).

A literature search of PubMed, Embase, and Web of Science was performed for clinical studies on systemic corticosteroid versus vincristine therapies in treating KHE/TA. Pooled relative risks (RRs) and response rate with 95% confidence intervals (CIs) were used to measure outcomes. Heterogeneity, subgroup analysis, sensitivity analysis, and publication bias analysis were performed for result evaluation.

Thirteen studies, comprising 344 participants, were used in the analysis. Vincristine therapy was found to be relatively more effective than systemic corticosteroids (RRs = 0.45, 95%CI: 0.35–0.58). The result of pooled adverse reactions response rate for systemic corticosteroids was 0.31 (95%CI, 0.18–0.43), significantly higher than that for vincristine, which was 0.12 (95%CI, 0.06–0.19). In subgroup analyses, factors including mean age and race of patients, and period of follow-up were examined as possible sources of heterogeneity.

This is the first meta-analysis estimating the clinical outcomes of systemic corticosteroids in comparison with those of vincristine in the treatment of KHE/TA. The results showed that vincristine was considerably more effective with lower complication rates than systemic corticosteroids; thus, vincristine could be suggested as the first-line therapy for KHE/TA.

## INTRODUCTION

Kaposiform hemangioendothelioma (KHE) and tufted angioma (TA), first discovered by Zuckerberg in 1993, are vascular neoplasms, which usually present with expanding erythematous or violaceous soft tissue masses.^1^ Unlike infantile hemangiomas, KHE and TA have distinct histological features including infiltrating nodules, spindle-shaped cells, and slit-like vascular channels.^2^ Although most cases involve the skin, KHE and TA are locally aggressive and frequently associated with the Kasabach–Merritt phenomenon (KMP), a life-threatening coagulopathy, which is characterized by platelet trapping within an enlarging vascular tumor.^3,4^ For many years, a number of therapies have been proposed for the treatment of KHE/TA, but without a consistent outcomes.^5^ Moderate- to high-dose glucocorticoids were considered the main therapy for treating KHE/TA^2^; however, these steroids are associated with poor response rates and multiple short-term/long-term side effects. Subsequent studies have reported vincristine, a natural vinca alkaloid isolated from the leaves of periwinkle as another optimal treatment for KHE/TA.^5^ Over the course of time, vincristine has been recommended as an alternative first-line agent for treating KHE with KMP owing to its efficacy and safety profile.^6^

The aim of this meta-analysis was to compare systemic corticosteroids therapy with vincristine therapy in terms of efficacy and side effects in the treatment of KHE/TA.

## MATERIALS AND METHODS

The study protocol used was in accordance with recommendations of the Cochrane Collaboration and the PRISMA guidelines.^7^

### Search Strategy

An electronic search (from 1997 to 2015) was conducted to identify studies on systemic corticosteroids versus vincristine in treating KHE/TA by using PubMed, Embase, and Web of Science.^8^ The following terms were used in the literature search: tufted angioma, kaposiform hemangioendothelioma, Kasabach–Merritt phenomenon, systemic corticosteroids, and vincristine. The references used in the selected studies were also searched for prospective studies.

### Inclusion Criteria

Studies meeting the following criteria were included in the analyses: (1) studies using the human subjects; (2) studies on KHE/TA with/without KMP; (3) comparative studies of systemic corticosteroids versus vincristine treatment.

### Exclusion Criteria

A study was excluded from the analysis if it was: (1) conducted *in vitro*/in a laboratory; (2) a letter or a review; (3) an abstract only.

### Study Selection and Data Extraction

The titles and abstracts of the selected studies were screened by 2 reviewers (XHL and JYL) independently, and then a full text evaluation was performed according to the inclusion and exclusion criteria. The following data were extracted: last name of the first author, year of publication of study, number of subjects, subject's race, age of subject in months, treatment protocol, data on therapy response, side effects, and length of follow-up period. Any discrepancies in study selection and data extraction were discussed with a third investigator (JWZ, with >30 years of experience in treating KHE/TA; and XHQ, with >10 years of experience in statistical analysis).

### Quality Assessment

Quality of the studies was assessed by using the Strengthening the Reporting of Observational Studies in Epidemiology (STROBE) checklists. Twenty-two items relevant to the quality assessment appraisal were used. Scores ranging from 0 to 20 were defined as low to high quality, respectively.^9^

### Data Analysis

The risk ratios (RRs) with a 95% confidence interval were pooled to evaluate associations in the effect of corticosteroids versus vincristine across studies. For binary outcomes, the pooled average complication rate of 2 therapies was also calculated. Heterogeneity was assessed by using the Chi-square distributed. The Cochrane *Q* test and formally quantified by *I*^2^ statistics: (*I*^2^≤25%: low, 25% < *I*^2^ < 50%: moderate, *I*^2^≥75%: high), with *P* value < 0.05 indicating statistical significance.^10,11^ A random-effects model (if significant heterogeneity was detected) or a fixed-effects model was used in the data analyses.^12^ Subgroup analyses were also conducted to identify the independent variables (i.e., number of participants, age of patients, period of follow-up, and race of participants). Forest plots combined with funnel plot, and Egger's and Begg's regression tests were calculated to measure outcomes and to detect publication bias, respectively.^13^ Sensitivity analyses were also performed to quantitatively assess heterogeneity.

R software 2.13.0 meta package (Institute for Statistics and Mathematics) was used for between-study data analyses.

All analyses were based on previous published studies; thus, no ethical approval and patient consent are required.

## RESULTS

### Search Results

**FIGURE 1:**
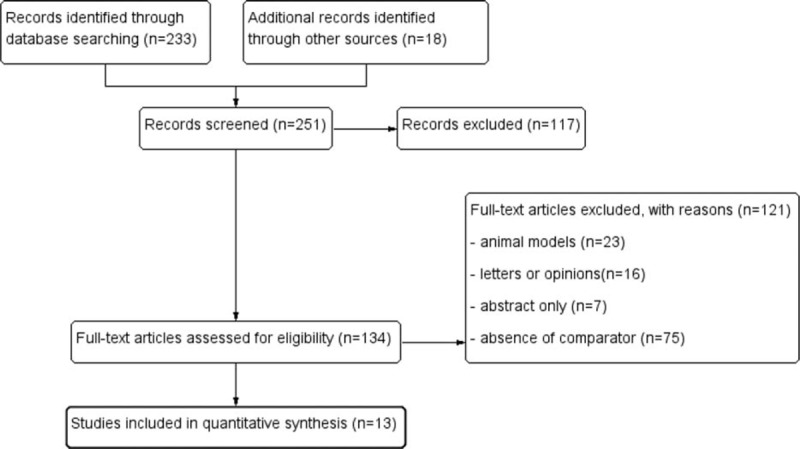
Flowchart of study selection process.

Figure 1 shows the flowchart of the selection process used in the study. A total 251 studies were identified from published data and were separately assessed by 2 reviewers. Also, 117 studies were excluded after evaluating titles and abstracts evaluations, and a further 121 studies were excluded after full text assessments (animal models = 23, letters or opinions = 16, abstract only = 7, and absence of comparator = 75). In the final analysis, 13 studies^3,5,14–24^ met the inclusion criteria and were used included. Observers reached an agreement on the studies included.

### Study Characteristics

The study characteristics are summarized in Table 1. Thirteen eligible studies^3,5,14–18,20–24^ published between 1997 and 2015 were included in the meta-analysis. The mean age of 344 participants was 3.61 months (from 0.9 to 11 months). Studies were conducted across different geographic locations (United States = 6, Europe = 3, and Asia = 4). The mean duration of the studies was 3.73 years (varing from 1 year to 18 years). Nine^3,5,14,15,18,20,22–24^ of the 13 studies reported side effects. The average study quality was 15.38 (range 9–17.5) on a scale of 0 to 20, as evaluated using the STROBE score.

**TABLE 1:**
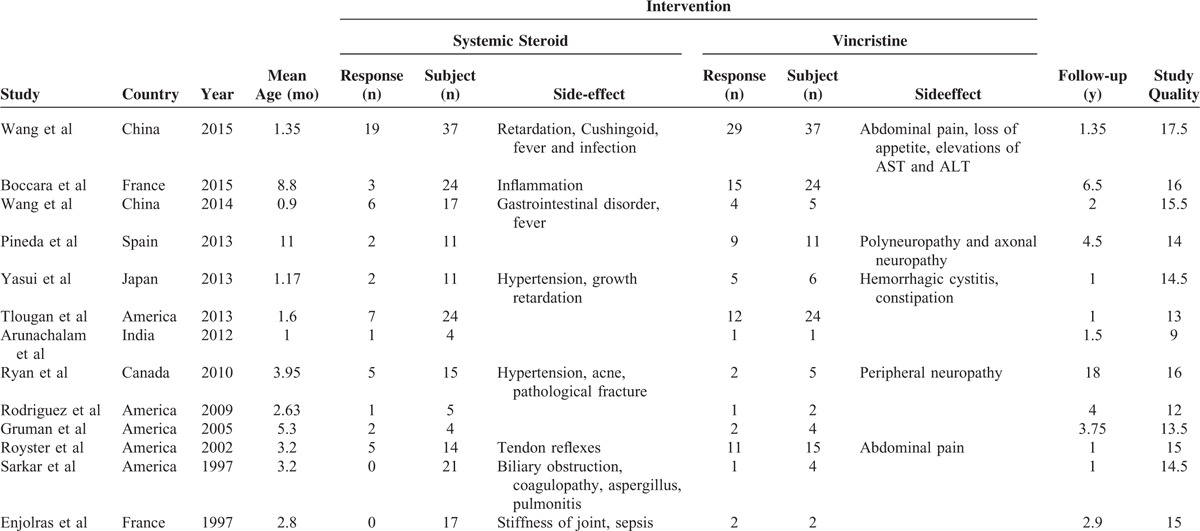
Characteristics of the Included Studies

### Systemic Corticosteroids Versus Vincristine

**FIGURE 2:**
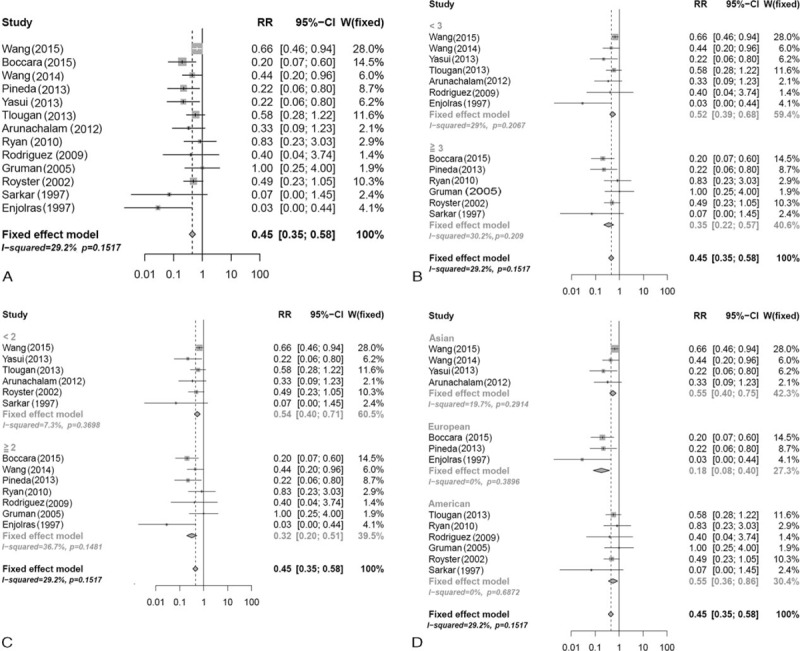
Forest plot of the effectiveness of systemic corticosteroids versus vincristine according to: (A) overall effect; (B) age of patients; (C) period of follow-up; (D) race of participants.

Thirteen studies^3,5,14–24^ with a total of 344 participants compared the effect of systemic corticosteroids with that of vincristine. The pooled results indicated that the effect of vincristine was relatively better than that of systemic corticosteroids (RR = 0.45, 95%CI: 0.35–0.58), with lower heterogeneity among the studies (*I*^2^ = 29.2%, *P* = 0.15) (Figure 2A).

In the subgroup analyses (Table 3), RR was 0.35 (95%CI, 0.22–0.57) for participants aged ≥3 months and 0.52 (95%CI, 0.39–0.68) for participants aged < 3 months (Figure 2B); 0.32 (95%CI, 0.02–0.51) for the follow-up period ≥2 years compared to 0.54 (95%CI, 0.40–0.71) for the follow-up period < 2 years (Figure 2C); 0.55 (95%CI, 0.36–0.86) for American, 0.18 (95%CI, 0.18–0.40) for European, and 0.55 (95%CI, 0.40–0.75) for Asian participants (Figure 2D).

**TABLE 3:**
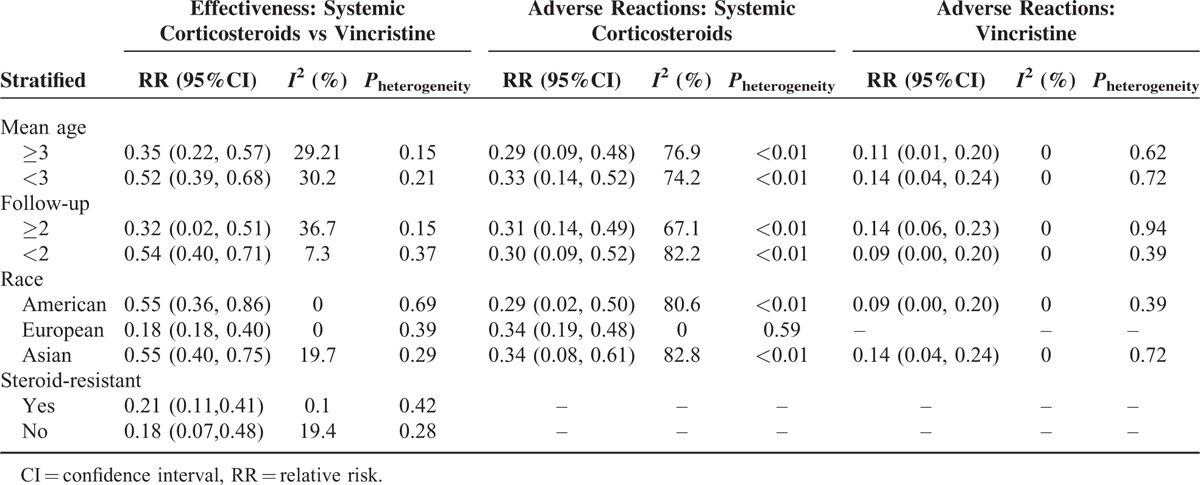
Subgroup Analysis of Systemic Corticosteroids and Vincristine

Six studies^3,5,18,20,22,23^ recorded that patients had steroid-resistant KHE/TA. The result for steroid-resistant cases was 0.21 (95%CI, 0.11–0.41) in contrast to 0.18 (95%CI, 0.07–0.48) for the nonresistant ones.

### Adverse Reactions

**FIGURE 3:**
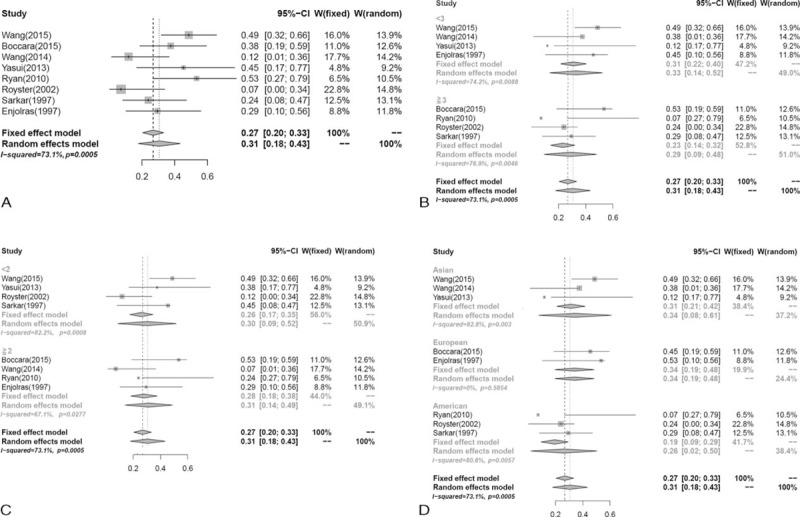
Forest plot of the adverse reactions of systemic corticosteroids according to: (A) overall effect; (B) age of patients; (C) period of follow-up; (D) race of participants.

Eight studies^3,5,14,15,18,22–24^ reported adverse reactions with the use of systemic corticosteroids including Cushingoid appearance (n = 15), hypertension (n = 8), fever and infection (n = 10), retardation (n = 2), inflammation (n = 10), gastrointestinal disorder (n = 1), pathological fracture (n = 1), tendon reflexes (n = 1), biliary obstruction (n = 1), coagulopathy (n = 2), and stiffness of joint (n = 2) (Table 2). The pooled result was 0.31 (95%CI, 0.18–0.43). There was a relatively high heterogeneity observed among the studies (*I*^2^ = 73.1%, *P* = 0.0005) (Figure 3A).

**TABLE 2:**
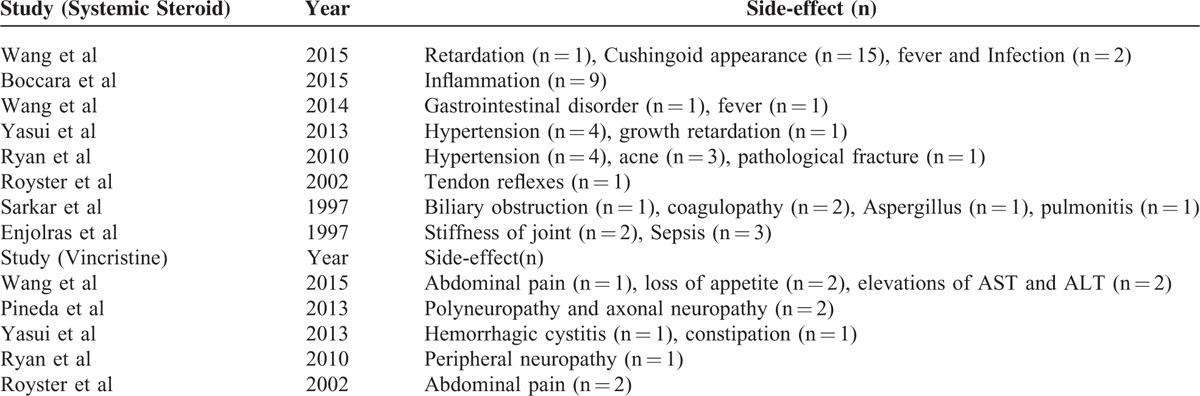
Adverse Reactions of Systemic Corticosteroids and Vincristine

In the subgroup analyses (Table 3), the pooled response rate of systemic corticosteroids was 0.29 (95%CI, 0.09–0.48) for participants aged ≥3 months and 0.33 (95%CI, 0.14–0.52) for participants aged < 3 months (Figure 3B); 0.31(95%CI, 0.14–0.49) for the follow-up period ≥2 years compared to 0.30 (95%CI, 0.09–0.52) for follow-up period < 2 years (Figure 3C); 0.26 (95%CI, 0.02–0.50) for American, 0.34 (95%CI, 0.19–0.48) for European, and 0.34 (95%CI, 0.08–0.61) for Asian participants (Figure 3D).

**FIGURE 4:**
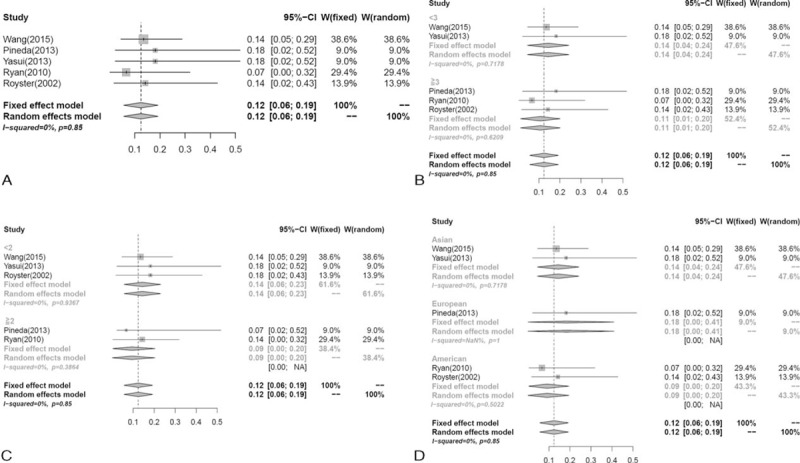
Forest plot of the adverse reactions of vincristine according to: (A) overall effect; (B) age of patients; (C) period of follow-up; (D) race of participants.

Five studies^3,5,18,20,22^ described side effects after treatment with vincristine including neuropathy (n = 3), abdominal pain (n = 3), hemorrhagic cystitis (n = 1), constipation (n = 1), loss of appetite (n = 2), and elevations of aspartate aminotransferase (AST) and alanine aminotransferase (ALT) levels (n = 2) (Table 2). The pooled result was 0.12 (95%CI, 0.06–0.19). There were no statistically significant differences in heterogeneity (*I*^2^ = 0%, *P* = 0.85) (Figure 4A).

In the subgroup analyses (Table 3), the pooled response rate of vincristine was 0.11 (95%CI, 0.01–0.2) for participants aged ≥3months and 0.14 (95%CI, 0.04–0.24) for participants aged < 3 months (Figure 4B); 0.14 (95%CI, 0.06–0.23) for the follow-up period ≥2 years compared to 0.09 (95%CI, 0.00–0.2) for the follow-up period < 2 years (Figure 4C); 0.09 (95%CI, 0.00–0.20) for American and 0.14 (95%CI, 0.04–0.21) for Asian participants (Figure 4D).

### Sensitivity Analysis

In this meta-analysis, similar results were obtained among the studies. Sensitivity analyses demonstrated that the exclusion of studies from the pooled analyses did not influence the results obtained.

### Publication Bias

**FIGURE 5:**
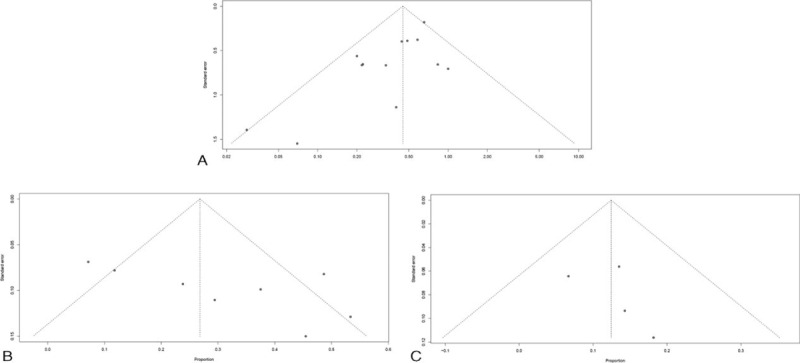
Funnel plot of standard error by standard differences in: (A) the effectiveness of systemic corticosteroids versus vincristine; (B) adverse reactions of systemic corticosteroids; (C) adverse reactions of vincristine.

Considering the effects of systemic corticosteroids versus vincristine (Begg's test *P* = 0.11; Egger's test *P* = 0.01), no evidence of publication bias was found. Given the limited number of included studies, a small publication bias in the adverse reactions resulting from systemic corticosteroids was determined by funnel plot visualization (Figure 5).

## DISCUSSION

The present meta-analysis showed that vincristine was relatively more effective and associated with a lower complication rate in the treatment KHE/TA than systemic corticosteroids.

KHE/TA is a vascular tumor often accompanied by profound lymphangiomatosis and thrombocytopenia. According to Sarkar et al, lesions usually emerge at birth or in early infancy and are associated with a typical indurated red plaque on the extremities, trunk, and sometimes head and neck.^15,16^ Steroids have been used and remained as the first-line treatment for KHE/TA for several decades. According to Drolet, prednisolone at a dose of 2 mg kg^−1^ d^−1^ was established as the consensus-derived treatment protocol.^25,26^ However, in contrast to its wide use, patients on steroid monotherapy showed poor response, and/or recurrences when therapy was stopped. In the study by Enjolras et al,^14^ none of the 25 patients studied showed a good response to steroids. In our meta-analysis, 26% of patients responded to systemic corticosteroids treatment.

In recent studies, vincristine as a pharmaco-therapeutic agent was reported to have a relatively high response rate in treating KHE/TA with KMP. Isolated from periwinkle's (*Catharanthus roseus*) leaf, vincristine is an alkaloid that can inhibit mitosis in microtubules.^27^ Haisley-Royster et al^5^ studied 15 patients with KMP treated with vincristine and observed an increase in platelet count of at least 20 × 10^9^ in all cases soon after treatment. A recent retrospective study of vincristine conducted by Wang et al,^3^ however, found an improvement in 78% of the patients studied. And in this study, the response rate for vincristine therapy was 66%. There are different opinions on the effect of treatments for KHE/TA. Yoon et al^28^ have reported of a successful case treated with a combination of steroid, interferon-alpha, and vincristine. Our analyses included a study by Fernandez-Pineda et al,^20^ which showed a stable outcome of vincristine–aspirin–ticlopidine therapy for vascular tumors (KHE/TA) associated with KMP. Owing to the lack of sufficient data, a corresponding conclusion cannot be made. Meanwhile, a subgroup analysis of steroid-resistant cases yielded better outcomes than the results from nonresistant cases. Based on aforementioned studies, it can be said that vincristine is useful in steroid-resistant cases and is comparatively more efficacious in treating KHE/TA.

According to Pandey et al,^29^ long-term treatment with highdose systemic corticosteroids was reported to cause multiple adverse reactions (e.g., Cushing syndrome, cataract, diabetes, hypertension, myopathy, osteoporosis, and infection). According to our research, 53 patients in 8 studies experienced with side effects during/after corticosteroids treatment, including cushingoid appearance (28.3%), infection (18.9%), inflammation (18.9 %) among others. Following the long follow-up period after vincristine treatment, fewer complications^30^ such as cellulitis, peripheral neuropathy, constipation, ileus, SIADH, seizure, leukopenia, and myeloid suppression were reported. In our analysis, 12 patients in 5 studies of vincristine treatment developed symptoms such as reversible neuropathy (25%) and abdominal pain (25%), most of which were transient. The pooled response rate was 0.31 (95%CI, 0.18–0.43) and 0.12 (95%CI, 0.06–0.19) for systemic corticosteroids and vincristine treatments, respectively. Compared with traditional corticosteroids therapy, vincristine treatment yielded better outcomes.

To our knowledge, this is the first meta-analysis comparing the effect of systemic corticosteroids with that of vincristine therapy in the treatment of KHE/TA and evaluating the safety of these 2 therapies. Subgroup analyses were also assessed for possible sources of heterogeneity, with factors including mean age and race of patients, and period of follow-up. The results showed that vincristine therapy appears to be a safer and more effective treatment option than systemic corticosteroids. In addition, better outcomes were observed inpatients <3 years with fewer complications than older patients, thus providing a strong evidence for supporting the necessity of early intervention in KHE/TA cases.

This meta-analysis has some potential limitations: (1) the sample size of each trial was relatively small; (2) several studies of treatments for KHE/TA were suggested for systemic steroid-resistant cases, which might limit the accuracy of the results of our study; (3) the quality of individual study was varied, some having limited adjustment for potential statistical confounding; and (4) there were some methodological drawbacks in the selection of the studies.

Several questions remain to be answered. According to previous studies, patients responded to moderate- to high-dose glucocorticoids, and long-term use of corticoids at high doses results in serious side effects. As a result, the relationship between therapy dose, and the response rate or the complication rate remains unknown. Recently, 2 studies^20,28^ mentioned the use of combination therapy. Would the concomitant use of systemic corticosteroids and vincristine result in a different outcome? To answer this question, further studies including well-designed randomized controlled clinical trials with adequate control for confounding factors should be considered.

In conclusion, the result of this meta-analysis showed that vincristine was relatively more effective in treating KHE/TA with a lower complication rate than systemic corticosteroids; thus, vincristine could be suggested as the first-line therapy for KHE/TA.
